# Modelling event-related skin conductance responses

**DOI:** 10.1016/j.ijpsycho.2010.01.005

**Published:** 2010-03

**Authors:** Dominik R. Bach, Guillaume Flandin, Karl J. Friston, Raymond J. Dolan

**Affiliations:** Wellcome Trust Centre for Neuroimaging, University College London, 12 Queen Square, London WC1N 3BG, United Kingdom

**Keywords:** Skin conductance, SCR, Galvanic skin response, GSR, Electrodermal activity, EDA, Convolution, Deconvolution, General linear model, Linear time-invariant filter, Biophysical models

## Abstract

Analytic tools for psychophysiological signals often make implicit assumptions that are unspecified. In developing a mathematical framework for analysis of skin conductance responses [SCRs], we formalise our assumptions by positing that SCRs can be regarded as the output of a linear time-invariant filter. Here, we provide an empirical test of these assumptions. Our findings indicate that a large component of the variance in SCRs can be explained by one response function per individual. We note that baseline variance (i.e. variance in the absence of evoked responses) is higher than variance that could not be explained by a linear time-invariant model of evoked responses. Furthermore, there was no evidence for nonlinear interactions among evoked responses that depended on their temporal overlap. We develop a canonical response function and show that it can be used for signals from different recording sites. We discuss the implications of these observations for model-based analysis of SCRs.

## 1 Introduction

The essence of psychophysiology is to infer psychological processes from measured physiological signals. Rendering such inference plausible rests on assumptions about how these signals are generated, albeit in many instances without a formal specification. For example, the amplitude of event-related skin conductance responses [SCRs] is used to infer sympathetic arousal, where we know that SCRs are generated by sweat secretion initiated by distinct bursts of sudomotor nerve activity (Boucsein, 1992). These sudomotor firing bursts directly relate to autonomic arousal, but the amplitude of the ensuing SCRs is only informative if there is a (linear) mapping from sudomotor firing (and hence, arousal) to SCR amplitude. The most parsimonious biophysical system that produces such a mapping would generate SCRs that are scaled versions of a template. Also, when two responses overlap, it is frequently posited that if some baseline can be estimated for the second response, its amplitude is not affected by the preceding response (Boucsein, 1992; Barry et al., 1993; Lim et al., 1997; Alexander et al., 2005). This amounts to assuming that the compound response is simply the sum of two single responses.

Model-based analysis for SCR has been proposed for responses that occur in rapid succession (Barry et al., 1993; Lim et al., 1997; Alexander et al., 2005; Bach et al., 2009; Benedek and Kaernbach, in press). The use of explicit mathematical models (Bach et al., 2009) makes it necessary to formalise the underlying assumptions. The advantage of such explicit models is that assumptions underlying both classical and model-based methods are fully specified and can thus be tested. Here, we provide a test of the aforementioned assumptions about SCRs in order to show the validity of our model-based approach. Although similar assumptions have been made in previous approaches, they have not been tested formally.

We assume that SCRs are generated by a linear time-invariant [LTI] system (Bach et al., 2009), a standard concept in signal processing. Time-invariance in this context corresponds to saying that within any individual and experimental condition, SCRs are scaled versions of a template. Linearity means that the response to any number of events equals to the sum of responses to each individual event. To substantiate these assumptions, we have shown that (a) under the linearity assumption, SCRs can be deconvolved even at inter stimulus intervals [ISIs] as short as 3 s, and (b) that in non-overlapping responses, about 75% of the total signal variance can be explained by one impulse response function, where the residual variance incorporates noise and spontaneous fluctuations. Finding (a) suggests that violations of the linearity assumption do not necessarily compromise analysis but does not provide a rigorous test of linearity. Likewise, while finding (b) suggests that the impulse response function can be regarded as largely time-invariant, it does not provide a quantitative estimate of violations of time-invariance. This is because the residual variance could not be partitioned into noise and variations in the response function per se. Furthermore, we have suggested that the time-invariance property generalises to a stereotyped response across individuals and stimulus types; thus making the use of a canonical response function feasible. We have however only tested two stimulus classes, that is, aversive white noise and negative, arousing (i.e. aversive) pictures, and used recordings from one electrode site (thenar/hypothenar). Here, we test linear and time-invariance assumptions in a more rigorous way by (a) quantifying resting-state signal variance in relation to residual variance in event-related responses; (b) testing the time-invariance assumption for more classes of stimuli; (c) testing linearity by presenting paired stimuli and comparing repetition effects at different ISIs; and (d) generalising these findings to other recording sites.

Event-related SCRs in various experimental contexts are summarised in Boucsein (1992) and can be grouped into different classes: (a) phasic orienting responses to simple stimuli, often elicited by white noise sounds with loudness between 20 dB and 100 dB, usually requiring a motor reaction; (b) defensive reactions to potentially harmful stimuli, e.g. elicited by sounds around 100 dB loudness; and (c) responses to stimuli requiring more complex information processing and reflecting stimulus significance, e.g. by experimental instructions, or per se, for example when viewing emotional pictures. We have previously tested the time-invariance of responses to simple loud noises [category (a)] and emotional picture viewing [category (c)]. Here, we sought to test potentially harmful stimuli that fall into category (b), and responses to stimuli that are rendered significant by experimental instructions. Therefore, we measured responses to electric shocks slightly below the pain threshold, and to targets in an auditory oddball and a visual detection paradigm. We report data from these experiments, together with responses to single white noise bursts and compare them with responses from two experiments published previously (Bach et al., 2009).

As we sought to quantify baseline variance of the skin conductance signal we needed a context where baseline responses are comparable to spontaneous fluctuations during evoked responses. In pilot experiments, we observed that baseline activity decreases rapidly when no stimulus is present for more than 30–40 s. Therefore, to obtain a comparable estimate of spontaneous fluctuations in the presence and absence of evoked responses, we chose to measure these in the visual detection paradigm; where a continuous stream of stimuli is presented, only one of which is to be attended. During the baseline period, the distractor stimuli were presented, but no target occurred. We hoped to show that spontaneous fluctuations (as indexed by variance in the absence of target stimuli) were a sufficient explanation for the residuals of a time-invariant model of evoked responses. In other words, we hoped to show that the baseline variance was equal or greater than the residual variance, under the assumption that the shape of the evoked responses did not vary from trial to trial.

In order to test the linearity assumption, we presented two stimuli with an inter stimulus interval [ISI] of 2, 5.5, or 9 s. Expected responses were estimated from responses to single stimuli during separate trials. Nonlinear responses imply that the SCR to the second stimulus depends on the response to the first, provided they occur sufficiently close together. This attenuation of the second response as a function of the first depends on the elapsed time (ISI) between them. Thus, it represents a repetition × ISI interaction. We expected to see a simple repetition effect (that can be attributed to adaptation of the underlying neuronal response) but were more interested in the interaction with ISI, which should be negligible under linear (i.e., superposition) models.

To ensure that the results obtained in these tests are not confined to palmar (thenar/hypothenar) recordings, we conducted a further experiment with simultaneous recordings from palm, fingers, and foot, in order to compare explained variance and the response function across recording sites.

In addition to testing model assumptions, we were able to develop a refined canonical response function (CRF), based on 1278 SCRs from 64 individuals, and analytically modelled as an exponentially modified Gaussian function. Similar functions have been used previously for example in modelling chromatographic peaks (for a review see e.g. DiMarco and Bombi, 2001) and nutrient uptake in mammary glands (Qiao et al., 2005). This synthetic CRF is now included in the functions scr_bf_crf and scr_bf_infbs in the Matlab software *SCRalyze* that has been released under the *GNU General Public License* and is obtainable from http://scralyze.sourceforge.net*.*

## 2 Methods

### 2.1 Participants

We recruited healthy unmedicated participants from the general population who all received monetary compensation for participation. Twenty individuals (10 male, 10 female, mean age ± standard deviation: 21.8 ± 3.3 years, range 19–30 years) took part in experiments 1 and 2, and 22 individuals (11 male, 11 female, mean age ± standard deviation: 24.7 ± 4.5 years, range 19–34 years) participated in experiments 3 and 4. Both subject samples were independent of each other and those in previous experiments (Bach et al., 2009). Twenty-six participants, partly overlapping with previous experiments, took part in experiment 5 (12 male, 14 female, mean age ± standard deviation: 24.4 ± 4.9 years, range 19–35 years). All participants gave written informed consent, and the study was approved by the local ethics committee.

### 2.2 Stimuli and apparatus

#### 2.2.1 Experiment 1

We used 10 uncomfortable electric shocks to elicit SCRs, delivered via a pin-cathode/ring-anode configuration attached to the dominant forearm as a 500 Hz current train with individual square pulse width of 0.5 ms, varying current amplitudes (mean ± SD: 0.78 mA ± 0.43 mA) for 100 ms. Before the experiment, discomfort and pain thresholds were assessed with increasing stimulation intensity, with stimulation intensity being set as just below the pain threshold. Two epochs from one participant were missing due to a faulty trigger. This experiment always preceded experiment 2 in order to avoid spontaneous responses associated with prolonged anticipation of electric shocks.

#### 2.2.2 Experiment 2

This experiment used an auditory oddball task with 10 oddball stimuli. Every second, one of two sine tones (50 ms length; 10 ms ramp; ∼ 75 dB; 440 or 660 Hz, respectively) was delivered via headphones (PX-660 Pro Luxe, Fujikon, Hong-Kong, China). The participant was instructed to press a computer key on hearing the oddball tone, the pitch of which was balanced across participants.

#### 2.2.3 Experiment 3

In this visual detection task, a white letter was flashed on a black screen for 200 ms with 800 ms blank intervals. A red cross target stimulus was embedded in this stream and participants were asked to press a computer key when they detected the target. An additional baseline period of 60 s without targets was added to the beginning or end of the experiment (balanced across participants). To habituate participants to the distractor stimuli, each part of the experiment was preceded by 20 distractor stimuli. This experiment always preceded experiment 4, which we thought would be less prone to habituation.

#### 2.2.4 Experiment 4

This experiment involved white noise sounds in four experimental conditions: single stimuli and double stimuli with an ISI of 2 s, 5.5 s, or 9 s. To avoid different subjective expectations about subsequent stimuli (between the first and the second stimulus), we additionally presented triple stimuli with ISIs randomly determined to be 2 s, 5.5 s, or 9 s. These trials were not analysed. For each experimental condition and for the triple stimuli, 10 trials were realised, summing to 50 trials in randomised order, and 100 sound stimuli. These were white noise sounds of 1 s length (10 ms onset and offset ramp, ∼ 85 dB sound pressure level), delivered via headphones (PX-660 Pro Luxe). Participants were instructed to press a button on a computer keyboard as quickly as possible when they heard a sound. A fixation cross was visible on the screen all the time. The first trial was preceded by 2 s of silence, and the last stimulus of each trial was followed by 30, 35, or 40 s of silence.

#### 2.2.5 Experiment 5

The same stimulus type and equipment as in experiment 4 were used. Participants heard 20 single white noise bursts over headphones, and participants were asked to make a response to each sound with a pedal for the dominant foot.

#### 2.2.6 Common settings

For experiments 1–3 and 5, ISI was randomly chosen to be 29 s, 34 s, or 29 s, with a mean of 34 s for each participant. All experiments were programmed in Cogent (Version 2000v1.25; www.vislab.ucl.ac.uk/Cogent) on Matlab 6.5 (MathWorks; Natick MA; USA), and run on a personal computer with a Pentium 4 processor and a SoundMax soundcard (Analog Devices, Norwood MA, USA).

Skin conductance was recorded on the thenar/hypothenar of the non-dominant hand using 8 mm Ag/AgCl cup electrodes (EL258, Biopac Systems., Goleta CA, USA) and 0.5%-NaCl electrode paste (GEL101; Biopac Systems). In experiment 5, additional recordings were made from the volar middle phalanx of the dominant 2nd/3rd finger, and the medial plantar surface of the non-dominant foot as described in Boucsein (1992, p.99) Recordings were conducted in a magnetically shielded room (MSR), using a custom-built constant voltage coupler (2.5 V), based on a differential amplifier and DC-powered by a 12 V battery to minimise electromagnetic noise. The output of the coupler was converted into an optical pulse frequency. This varies sampling rate over time, such that the effective time resolution is determined by the lowest transmission frequency. The lowest sampling rates encountered in any participant were 93.9 Hz, 68.7 Hz, 24.0 Hz, 2.7 Hz, and 16.1 Hz, respectively for the five experiments (note that for the 5 participants with sampling rates smaller than 10 Hz in experiment 4, some aliasing might have been introduced during A/D conversion). This pulse signal was transmitted using fibre optics, digitally converted outside the MSR with 2 μs time resolution (Micro1401, Cambridge Electronic Design, Cambridge, UK), and recorded (Spike2, Cambridge Electronic Design, Cambridge, UK). Stimulus onset was signalled by TTL pulses of 10 ms length via the stimulus computer's parallel port, and recorded simultaneously with the same time resolution. Temperature and relative humidity of the experimental room were between 18–21.6 °C and 31–51% (experiments 1–2), 20.0–26.0 °C and 31–64% (experiment 3–4), and 21.6–27.6 °C and 45–68% (experiment 5).

### 2.3 Data analysis

Data analysis was carried out in Matlab 7.4 using custom code that is available from the authors. Prior to analysis, skin conductance data were converted back to a waveform signal with 100 Hz time resolution, filtered with a bidirectional first-order Butterworth bandpass filter and cut-off frequencies of 5 Hz, and 0.0159 Hz (corresponding to a time constant of 10 s), respectively, and down-sampled to 10 Hz sampling rate. The time series was then *z*-transformed to account for between-subjects variance in SCR amplitude, which can be due to peripheral factors such as skin properties (note that *z*-transformation for experiment 3 included both event-related and baseline responses). The 30 s following each event onset was extracted and analysed. In experiment 5, 14 out of 520 epochs were excluded due to recording equipment malfunction (i.e. periods where no data was recorded). To ensure a conservative estimate of residual variance, we did not exclude potential artefacts or non-responses. Note that this does not influence the shape of the estimated response function, as unsystematic noise would not be represented in the first principal component used to characterise the response function. Despite filtering, skin conductance level can differ between trials and consequently data from each trial was mean-centred. Baseline responses in experiment 3 was extracted during 60 s, divided into two periods of 30 s and analysed similarly. ANOVAs of parameter estimates were conducted in SPSS 14.0 (Chicago IL, USA). We report *ε*- and corrected *p*-values according to Greenhouse–Geisser.

## 3 Results

### 3.1 Test of the time-invariance assumptions

**Fig. 1:**
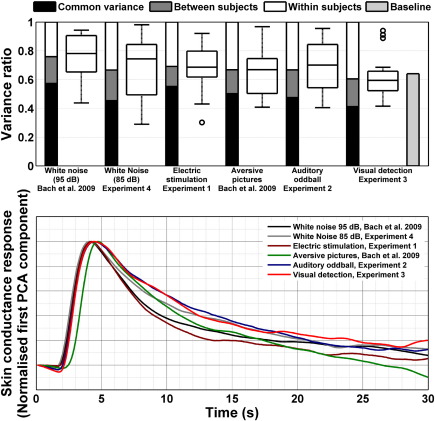
Variance partitioning and response functions were estimated for different stimulus classes. Top panel: bar charts show variance components across subjects; that is, variance explained by one common response function (black), between-subjects variance (grey) and residual variance (white) for each experiment. Light grey: baseline variance in the absence of evoked responses for the visual detection experiment. Box plots depict explained variance within subjects, showing median (line), quartiles (box), and range (whiskers). Outliers (values outside the 1.5 × interquartile range) are shown as individual dots; whiskers then represent the data within the interquartile range. Bottom panel: empirical (PCA) response functions across participants for the different experiments.

For each experiment and each participant, we performed a principal component analysis [PCA] of SCRs to determine the response function that explained the maximum variance, and quantify unexplained residuals. Results are summarised in Fig. 1. Across all experimental paradigms, the variance explained was above 60%, albeit with considerable between-subjects differences, as evident in the box plots. Thus, for some participants, almost all variance could be explained by one response function, whereas for others, the residual variance was as high as 70%. Across the group, baseline variance in the visual detection experiment amounted to 64% of the total variance during evoked responses, well above the corresponding residual variance.

The same procedure was then applied to the combined responses from all participants in each experiment. This enabled us to quantify the between-subject variance in response shape (note that this does not speak to the time-invariance assumption but quantifies how stereotyped the responses were across individuals). These response functions explained more than 40% of total variance for each experiment. The response functions are depicted in the lower panel of Fig. 1 and look similar, with the exception of a later peak for the aversive picture viewing experiment and a broader peak for the visual detection and auditory oddball experiment.

**Fig. 2:**
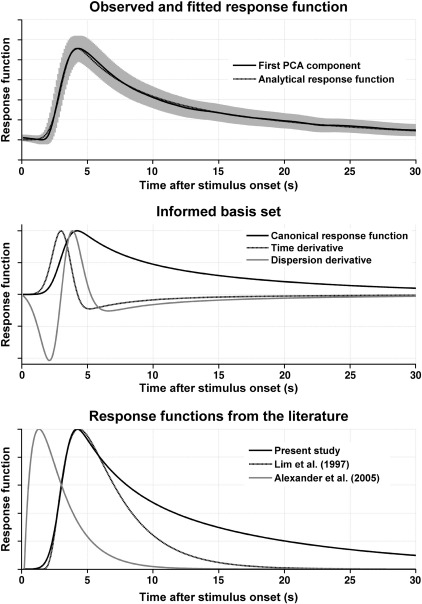
A canonical response function was derived from observed responses (shown as the first PCA component ± standard deviation across all observations), and was described analytically as an exponentially modified Gaussian function (top panel). Time and dispersion derivatives were constructed to account for differences in response shape between participants and experimental conditions (middle panel); the complete basis set could explain 64.0% of the total variance. Latency and rise time are comparable to the function proposed by Lim et al. (1997).

By combining all data, we created a canonical response function (CRF) based on 1278 SCRs from 64 participants, and a basis set, accounting for interindividual differences. The procedure is similar to the one used in Bach et al. (2009) and is described in Appendix A. The three basis functions of this set depicted in Fig. 2 explained 48.4%, 10.6%, and 5.0%, respectively, of the total variance, leaving 36.0% residual variance.

One issue to consider is how the high-pass filter influences the apparent response. High-pass filtering is necessary as we assume a finite response, but the raw skin conductance signal does not necessarily return to zero after an SCR. We therefore explored how the cut-off frequency of the high-pass filter impacts the explained variance within participants and experimental conditions. By applying filter frequencies between 0 and 0.025 Hz in 0.005 Hz steps, we show that across the whole group, the explained variance was estimated at 70% if no filter was applied and decreased, almost linearly, to 67.5% at the highest filter frequency. This shows that the filtering does not have an appreciable impact on the modelling of responses.

### 3.2 Test of the linearity assumptions

**Fig. 3:**
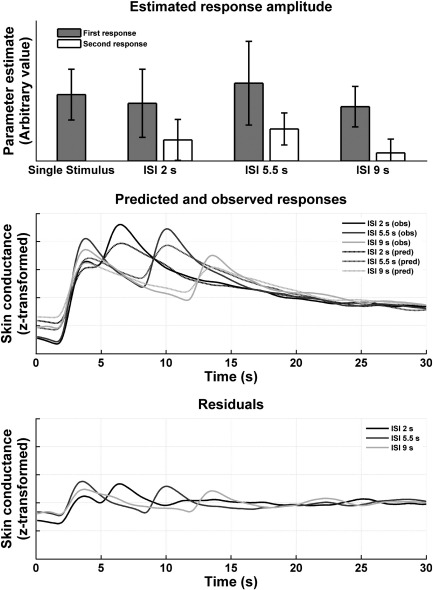
The linearity assumption was tested by presenting either single white noise sounds, or a sequence of two sounds with differing ISIs, separated by silent periods of 30–40 s. For each individual, a response function was estimated from their responses to single events, and responses to double events were estimated under time-invariance assumptions. It turns out that responses to second events are smaller than to the first event in each sequence, regardless of ISI (2–9 s); however, there is no evidence for dependency of the repetition suppression on ISI. Averaged residuals are similar for the first and second event, thus indicating no systematic alterations of response shape at different ISIs.

For each participant from experiment 4, we determined a response function by performing PCA on responses to single stimuli. The first PCA component was then fitted to trials with double events by convolving it with two stick functions for each event onset, and combining this with a constant component. Parameter estimates controlling the height of the two stick functions are depicted in Fig. 3 and present the amplitude of the fixed-form responses for each trial type. Responses to the first (i.e. 30–40 s after the last stimulus) were larger than responses to the second sound, regardless of ISI; an effect that reached trend-level significance in a 2 (repetition) × 3 (ISI)-ANOVA (*F*_1, 21_ = 3.29; *ε* = 1; *p* = 0.08). When controlling for individual responsiveness by entering (mean-centred) single-response parameter estimates as a covariate, this effect was still significant at the trend level (*F*_1, 20_ = 3.89; *ε* = 1; *p* = 0.06). Additionally, individuals with higher responses to single stimuli had a higher repetition difference (*F*_1, 20_ = 4.82; *ε* = 1; *p* < 0.05). However, there was no main effect of ISI (all *F*s < 1.2) or interaction involving ISI (all *F*s < 1) in any of the analyses. This suggests that we were observing repetition suppression that did not depend on ISI or nonlinear interactions among stimulus-specific SCRs.

It is conceivable that within an individual, a refractory period scales with the amplitude of the first response, or in other words, that the second response is suppressed more when the first response is bigger. We tested this for each ISI separately and for all ISIs together by computing within-subjects regression slopes between the first and second response. A stronger suppression of the second after a bigger first response would imply negative regression slopes. The regression slopes were however positive across all ISIs (*t*_21_ = 4.4; *p* < 0.001). In other words, when the first response was relatively larger, the second was also increased. Within single ISIs, none of the regression slopes was significantly negative across subjects (all *p* > 0.80). There was no linear (*p* > 0.50) or quadratic (*p* > 0.05) relationship of the regression slope with ISI. Therefore, if there is any relation between first and second response within individuals, it is consistently positive, thus arguing against an amplitude-dependent refractory period.

In our previous study (Bach et al., 2009), we deconvolved the response function using an uninformed finite impulse response model. Parameter estimates from this model showed a time × ISI interaction, which might indicate a different response shape at different ISIs, but also overfitting of the data. In the present study, there was no indication of different response shape at different ISIs, such that our previous results can most likely be regarded as stemming from overfitting. Fig. 3 shows predicted and observed responses from the model described above. There is a systematic residual in the fit of the response peaks, but this does not differ between the first and the second response.

### 3.3 Generalisation to other recording sites

**Fig. 4:**
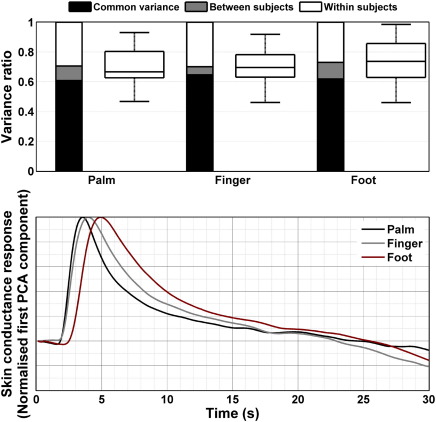
Variance portioning and response functions for hand (thenar/hypothenar), finger (volar surface, 2nd/3rd finger, middle phalanx), and medial foot (medial plantar) recordings. The ratio of explained variance is similar, and the response functions mainly differ in peak latency. Note that the canonical response function depicted in Fig. 2 can explain responses from all three recording sites almost equally well.

For experiment 5, we assessed time-lagged correlations between different recording sites across the complete unfiltered time series. Averaged across participants, palmar and finger recordings shared 50.3% variance at a time lag of 0.4 s, and palmar and foot recordings shared 30.4% at a time lag of 1.3 s. After filtering, PCA revealed similar within and between-subjects variance estimates and similar response functions for the recording sites as shown in Fig. 4. The mean peak latencies of the response functions were 3.9 s for palmar, 4.3 s for finger and 5.0 s for plantar recordings, thus closely resembling the lag values obtained for the whole, unfiltered time series. Across subjects, the latency differences between recording sites were significantly related to the distance between head and recording site. Between-subjects differences in this distance however had no effect on individual peak latencies into the predicted direction for any of the recording sites. Thus, it is not possible to predict the peak latency in one person's response function from the individual head/recording site distance.

The aforementioned CRF could explain on the average 50.0%, 53.7%, and 48.9% of the variance for palmar, finger, and foot recordings. The whole basis set explained 68.9%, 67.3%, and 66.0%. Adjusting the CRF to account for longer peak latencies in finger and foot recordings did not improve the amount of explained variance, possibly due to the fact that it was built from different experimental conditions, some of which already involved longer peak latencies than the ones obtained with white noise sounds. The estimated response amplitude was highest for palm recordings, followed by the finger (factor 0.60) and the foot (factor 0.33).

## 4 Discussion

In this paper, we tested the time-invariance and linearity of SCRs as these constitute two central assumptions for SCR analysis both in classical methods (Boucsein, 1992) and in model-based strategies, for example in a general linear convolution framework (Bach et al., 2009). We conclude that the time-invariance assumption seems to be met. While there was trend-level evidence for repetition suppression even at ISIs of 9 s, we found no evidence that this suppression depended on ISI and therefore no convincing evidence for nonlinear interactions among overlapping SCRs, although CNS mechanisms might lead to repetition suppression independent of peripheral factors.

We previously reported residual variance between 20% and 40% on orienting responses to loud noise and aversive pictures, and could extend this now to defensive reactions and responses to events that are rendered salient by experimental instruction alone. In a visual detection task, we included a baseline period without target stimuli that was used to estimate baseline fluctuations. This exceeded the residual variance during evoked responses by almost a factor two. That is, it appears that spontaneous fluctuations are suppressed when SCRs are elicited by external events. This suggests that a major component of residual variance during evoked responses stems from spontaneous fluctuations (i.e. the components that contribute to baseline variance). In other words, spontaneous or non-specific fluctuations are more than a sufficient explanation for variability in evoked SCRs, and the time-invariance assumption can be regarded as met for practical purposes. This generalises to recordings from foot and middle phalanx of the fingers where very similar results were obtained.

Across participants, the estimated response functions looked comparable in different experimental paradigms. On this basis, we were able to estimate a canonical response function (CRF), derived from 1278 SCRs from 64 individuals. This was extended to form an informed basis set that can account for between-subject differences in response shape, and for between-condition differences; such as slightly later responses to aversive pictures (see Fig. 2). In comparison with previously reported response functions (Lim et al., 1997; Alexander et al., 2005) our CRF has a much longer recovery time (see Fig. 2). It is however encouraging to note that our onset latency and peak are comparable with Lim et al. (1997). This is particularly important because their data were sampled at a fixed interval and adequately over-sampled, whereas we used a suboptimal (variable) sampling rate. The much shorter latency of the response function proposed by Alexander et al. (2005) is due to the fact that their function does not include the time due to sensory processing and sudomotor transmission (which occurs prior to the measurable SCR response). It is worth noting that our CRF can also be used for foot and finger recordings without compromising model fit, indicating that the between-subject variance in response shape within one recording site dominates over the variance caused by latency differences between recording sites. Finger and foot recordings might be advantageous as thenar/hypothenar recordings are prone to movement artefacts. Recordings from distal phalanges of the fingers have been reported in the literature, and although we only acquired data from the middle phalanx, one might tentatively speculate that the time-invariance property and the canonical response function also apply at the distal phalanx.

Also, we found the linearity principle seems to hold, providing one allows for categorical differences in the underlying neuronal response to an initial stimulus, relative to subsequent stimuli. Between 3 s and 10 s after an event onset, responses to a second event of the same type were attenuated. Although this effect was not statistically significant, it speaks to a classical repetition suppression that may be useful to model in some designs (see below). Crucially, however, there was no effect of ISI, suggesting that the SCR to the second stimulus does not depend on the overlap with the SCR to the first. This is consistent with a linear model, provided the model incorporates simple repetition effects. It seems plausible that this kind of repetition effect reflects central adaption to the stimulus (for an overview see Boucsein, 1992) but it could also be due to peripheral (e.g. sympathetic nerve) adaptation. Previous work (Bach et al., 2009) suggested that the response shape might be different at different ISIs but we could not confirm this in the present study. This does not rule out non-linearities in the SCR response function, but they are probably difficult to detect without a clear hypothesis about their form.

Repetition effects could be an issue for any form of SCR analysis, be it semi-visual or mathematical. Here, we suggest how they can be dealt with in the framework of a linear convolution model (Bach et al., 2009). If events engage a responsive system that has systematic variability, such that distinct levels of repetition are associated with different response amplitude, these levels can be encoded in different regressors (or, in classical analysis, in distinct levels of the experimental factor) (see Friston et al., 1998 for a discussion in the context of fMRI responses). Second, if there are systematic non-linearities in the responsiveness of the system, one can explicitly model these within the linear convolution framework using a Volterra series. Here, non-linearities are parameterised as coefficients of second and higher order Volterra kernels; together with the coefficients of the standard response function (first-order kernels). However, this is only necessary for more complex paradigms where there is no experimental control over repetition effects, or if we are specifically interested in such non-linearities, such as in studies of peripheral adaptation. Further work is needed to validate such models for the analysis of SCR and can be motivated by the fact that response habituation implies that the system we are modelling has memory.

An interesting possibility arises from the individual response functions depicted in Fig. 1, where aversive pictures evoke responses slightly later than other stimulus classes. There is no reason why the peripheral output system should exhibit a different response to one stimulus class than to any other. Given that images contain much more information than any other of the studied stimuli, this longer latency may reflect longer processing time in the central nervous system. The twist is that it might be possible to estimate characteristics of central nervous function by deconvolving the observed signal given a canonical response function. Recently proposed deconvolution/decomposition methods (Alexander et al., 2005; Benedek and Kaernbach, in press) have addressed this issue by attempting to recover the sudomotor time series; however they did not provide a model for how this sudomotor activity relates to processes in the CNS. We will pursue this in future work.

We emphasise that the present approach not only assists in analysis of SCRs, but also connects to the broader literature on psychophysiological responses and sets out a framework of how analysis assumptions and research questions can be formulated mathematically. For example, it has been proposed that a higher rate of biphasic as opposed to monophasic SCRs might differentiate defensive from orienting responses (Uno and Grings, 1965). In order to test this assumption, instead of visually scoring and counting responses (see Boucsein, 1992), the present framework allows one to parameterise condition-specific response functions and test for their differences.

In conclusion, we have investigated two central assumptions of SCR analysis that are usually not made explicit, but which are entailed for example in a general linear convolution framework (Bach et al., 2009). The time-invariance assumption could be supported, and there was no evidence for a violation of the linearity assumption, given repetition suppression is modelled. We suggest that repetition suppression does not preclude SCR analysis; indeed, non-linear methods can be incorporated into the linear framework, even if we do not have control over ISI. Thus, explicit modelling methods appear to be more powerful for SCR analysis than classical, semi-visual methods. Our findings pertain to many models that could be used for SCR. Indeed one might anticipate more refined models in the future, which might even relax the need for filtering the data prior to analysis. As noted by one of our reviewers, an interesting challenge here is to accommodate fluctuations in tonic skin conductance level, which can be substantially larger in amplitude than the SCR itself.

## Appendix A

In a previous paper, the canonical response function (CRF) was modelled as a Gaussian smoothed probability density function of a Gamma distribution (Bach et al., 2009). It turns out with the present dataset that the polynomial part of this function is estimated to be one, such that the CRF can be more parsimoniously described by a Gaussian smoothed exponential function, or exponentially modified Gaussian function. Using a bi-exponential function provided better fit across all single experiments and for the combined dataset than a mono-exponential function or other related functions, such as the exponential-Gaussian hybrid function used in chromatography (Lan and Jorgenson, 2001), or a (smoothed) sigmoid-exponential function as proposed by Lim et al. (1997). We now phenomenologically define the CRF as:

$${\tilde{h}}_{\text{can}}\left(t\right)=N\left(t\right)⊗\left(E_{1}\left(t\right)+E_{2}\left(t\right)\right)=\int {}_{-\infty }^{+\infty }N\left(t-\tau \right)\left(E_{1}\left(\tau \right)+E_{2}\left(\tau \right)\right)d\tau$$

where ⊗ denotes the linear convolution operator*, N*(*t*) is a Gaussian function with standard deviation *σ*, centred at *τ*

$$N\left(t\right)=\frac{1}{\sqrt{2\pi \sigma }}e^{-{\left(t-\tau \right)}^{2}/2\sigma^{2}}$$

and *E*(*t*) are exponential functions of the form

$$E_{1}\left(t\right)=e^{-\lambda_{1}t};E_{2}\left(t\right)=e^{-\lambda_{2}t}$$

The parameters of this analytical form of the CRF are estimated using the full dataset from this and a previous paper (Bach et al., 2009) with a least-square approach and gradient search. The estimated parameters are:

$$\left\{ \begin{array}{c} \hat{\tau }=3.0745\\ \hat{\sigma }=0.7013\\ {\hat{\lambda }}_{1}=0.3176\\ {\hat{\lambda }}_{2}=0.0708 \end{array}\right.$$

Note that the parameters have an approximate biophysical meaning, in the sense that *τ* defines the event-to-peak time, *σ* relates to the rise time, and the two exponential constants approximately define the decay.

We define an *informed basis set* {h̃_inf_^*i*^ (*t*)}_*i* = 1…*N*_, given that the CRF does not fit all individuals equally well. This basis set is generated by performing a multivariate first-order Taylor expansion of the CRF along time and dispersion parameters. Thus, for the construction of the temporal derivative $\frac{\partial {\tilde{h}}_{\text{can}}}{\partial t}$, the canonical function is differentiated with respect to time. The dispersion derivative $\frac{\partial {\tilde{h}}_{\text{can}}}{\partial \sigma }$ is obtained by computing the difference between the original CRF and the same one convolved with a Gaussian function of greater variance (*σ*′ = 1.2623).

The *informed basis set* capturing the CRF is therefore:

$${\left\{{\tilde{h}}_{\inf}^{i}\left(t\right)\right\}}_{i=1\ldots 3}=\left\{{\tilde{h}}_{\text{can}},\frac{\partial {\tilde{h}}_{\text{can}}}{\partial t},\frac{\partial {\tilde{h}}_{\text{can}}}{\partial \sigma }\right\}$$

This basis set was orthogonalised using a serial Gram–Schmidt process as implemented in the SPM software (spm_orth.m). The CRF is included in the *SCRalyze* software as function scr_bf_crf.m, and the basis set as scr_bf_infbs.m. The dataset on which this parameterisation was built is also included in the software distribution package.
